# Rejection of Schmidt et al.'s estimators for bear population size

**DOI:** 10.1002/ece3.5134

**Published:** 2019-04-24

**Authors:** Earl Becker, Aaron Christ

**Affiliations:** ^1^ Alaska Department of Fish and Game Anchorage Alaska; ^2^ U.S. Fish and Wildlife Service Homer Alaska

**Keywords:** bear estimation, distance sampling, rebuttal

## Abstract

Aerial distance sampling of bears to estimate population size has been used throughout many parts of Alaska. The distance sampling models are complex since they need to account for undetected bears and differences in detection probabilities. This will require covariates and mark‐recapture data. The models proposed by Schmidt et al. do not use covariates or mark‐recapture data and are inappropriate for these surveys.

## 1 INTRODUCTION

Advanced distance sampling, using small 2‐person aircraft, has been successfully used to estimate brown and black bear (*Ursus* spp.) population size in Alaska (Becker & Christ, 2015). Schmidt, Wilson, Thompson, and Reynolds (2017) presented a case for three alternative bear population estimators that can be applied to this data that they claim are less complex and more precise than the mark–recapture distance sampling (MRDS) methods used by Becker and Christ (2015). Their first model is a conventional distance sampling (CDS) model based on a half‐normal detection function (hereafter referred to as “CDS_Hn”). The second model pools data from several bear surveys into a hierarchical Bayesian CDS model that uses a half‐normal detection function (hereafter referred to as “CDS_HnBayes”), and estimates the population size of each study area. The third model is a Bayesian‐open population distance sampling model with a half‐normal detection function (hereafter referred to as “CDS_OpenHnBayes”). This model estimates *p*_a_, the proportion of the population in the searched area that is available to be detected to obtain the population estimate. All of these models use a half‐normal detection function with no covariates and do not utilize mark–recapture (MR) data. The first two models assume perfect detection for all bears, at a distance associated with the apex of detection, in order to obtain a population estimate. The half‐normal model discards distance data smaller than the distance of the detection apex, resulting in less data to fit the detection model. The lack of covariates causes all three models to assume all bears have the same probability of detection.

Unlike the three approaches proposed by Schmidt et al. (2017), the MRDS approach taken by Becker and Christ (2015) uses a two‐piece normal detection function to take advantage of the whole dataset to model both sides of the detection apex. They do not assume perfect detection at some fixed distance but rather estimate this parameter with a mark–recapture model at the distance associated with the apex of bear detection. In addition, covariates are used in both the distance (MCDS; Marques & Buckland, 2004) and the MR model to account for heterogeneity in bear detections. Combined these models form the MRDS model based on a 2‐piece normal distribution (hereafter referred to as “MRDS_2PN”). The MR model used within the MRDS_2PN model assumes “point independence” (Borchers, Laake, Southwell, & Paxton, 2006) between the pilot and backseat observer.

We agree with Schmidt et al. (2017) that estimating bear abundance and density is difficult. We also agree that model assumptions are important and that the MRDS_2PN model is complex. One main premise of Schmidt et al. (2017) is that the estimation bias from removing covariates and mark–recapture data is small and better inferences about changes in population size can then be made using their methods. They compare their model results with the “Becker‐Christ approach,” which is an MRDS model they created. Their “Becker‐Christ approach” model contains no covariates in either the distance model (two‐piece normal detection function) or the point independence MR model. MRDS models are not pooling robust, and for this reason, covariate modeling is very important in these models (Laake & Borchers, 2004). This lack of covariates in the “Becker‐Christ approach” creates a flawed MRDS model that is not equivalent to the MRDS‐PN model that uses covariates in modeling both the distance data and the MR data. Buckland, Rexstad, Marques, and Oedekoven (2015) consider MRDS models with no covariates (such as the “Becker and Christ approach”) to be so flawed that they do not consider them viable. For this reason, we disagree with the conclusions of Schmidt et al. (2017) that their models are “better” than those of Becker and Christ (2015). We feel the MR data and model cannot be eliminated and that covariates are required; they take a total error approach (Reynolds, 2012) that allows for biased estimates.

## 2 IS MARK–RECAPTURE DATA NEEDED?

The assumption of perfect detection on the transect line is listed as the most important assumption for CDS models (Buckland et al., 2001). For aerial surveys of brown and black bears in Alaska, that use small 2‐person aircraft with straight (non‐bubble) windows, maximum detection is achieved at a distance away from the transect line (Becker & Quang, 2009).

In determining if MR data are needed for distance sampling models, Borchers, Zucchini, and Fewster (1998) used the idea of certainty of detection on the transect (apex of detection in the case of bears) as the determining criteria. MRDS models are needed if any uncertainty exists; this has become a standard practice for distance sampling models (Borchers, Buckland, & Zucchini, 2002). Certainty about perfect detection at the detection apex is extremely difficult to obtain when aircraft is used. Alpizar‐Jara and Pollock (1996) noted that the assumption of perfect detection “is especially violated when using aerial surveys to estimate population size for marine mammals and other wildlife populations.” Early development of MRDS models was driven in part by developing models for aerially collected data. For aerially collected data, different MRDS models were developed by Manley, McDonald, and Garner (1996) for polar bears (*Ursus maritimus*), Quang and Becker (1996) for Pacific and common loons (*Gavua pacifica* and *G. immer*), Quang and Becker (1999) for brown bears (*Ursus *arctos), Becker and Quang (2009) for brown bears, and Becker and Christ (2015) for brown and black bears (*Ursus americanus*). These studies and others, such as (Laake, Dawson, & Hone, 2008), make it clear that mark–recapture data are needed to assess the perfect detection assumption when surveying with aircraft.

A cautionary tale about ignoring the perfect detection assumption is contained in the results of Laake's (1999) survey of a known population. Laake used distance sampling on a known population of 150 wooden stakes driven into the ground at random distances from and along a 1‐km transect in sage brush habitat of the western United States. This transect was independently walked and surveyed by 8 different observers. The assumption of perfect detection on the transect seems reasonable for this survey. We consider the assumption of perfect detection on the stake transect line to be more reasonable than perfect detection of all bears approximately 100 m (the detection apex) from an aircraft flying at 130 km/hr (80 mph) and 100 m above ground that frequently contains shrubs and trees. In the wooden stake data, the 8 CDS population estimates ranged from 85 to 164, the mean was 121 (*SD* = 37.67) resulting in a bias of −19.5% for the mean value. Using an MRDS model with point independence, the mean value was 146 (*SD* = 11.10) with a bias of −2.7% for the mean value. These results illustrate the importance of investigating the perfect detection assumption by fitting an MRDS model.

MRDS models use MR models with covariates to eliminate the assumption of perfect detection at the apex of detection (Laake & Borchers, 2004) needed by CDS models. Most MRDS models, including the Becker and Quang model (2009), assume all animals are detected independently between the two observers in the aircraft (pilot and observer) when modeling the MR data. Population estimates from MRDS models that assume full independence in detections between observers can have sizable bias, which is caused by the failure of the sampling process to meet the full independence assumption (Laake, 1999; Laake & Borchers, 2004). Borchers et al. (2006) proposed an MRDS model that assumes independent animal detections only at the apex of the detection function to deal with this problem; they labeled this assumption “point independence.” Their point independence MRDS models assume the MCDS model gets the right detection shape and adjusts the probabilities of the MCDS model with a mark–recapture model calculated at the distance at which detection is the highest. Laake and Borchers (2004) state that population estimates from MRDS models “obtained under the assumption of full independence will tend to be negatively biased compared to estimates obtained under the assumption of point independence.” The MRDS_2PN model uses an MR model that assumes point independence (Becker & Christ, 2015) between the pilot and observer.

Schmidt et al. (2017) justify dropping the MR model with the statement: “marginal detection probabilities at the apex of the detection function are quite high for both the pilot and observer (Becker & Christ, 2015; Becker & Quang, 2009; Walsh et al., 2010) suggesting that the joint *p*_d_ may approach 1.0 in many cases.” The MR probability estimates used to correct the distance detection probabilities will be different for full independence versus point independent MRDS models (Borchers et al., 2006; Laake, 1999). Un‐modeled heterogeneity in the MR model causes positive bias in the MR probability estimates and negative bias in the population estimate (Borchers et al., 2006; Buckland et al., 2015; Laake, 1999; Laake & Borchers, 2004). Using the stake data, an MRDS model with full independence had a lower population estimate (range: 76–142, mean = 113, *SD* = 17.85) than both the CDS model (mean = 121) and the MRDS model with point independence (mean = 146); this was caused by un‐modeled heterogeneity (Laake, 1999). To avoid this bias, Buckland et al. (2015) recommend the use of point independent MRDS models when the assumption of perfect detection is not met. We have observed this bias in numerous brown and black bear datasets (E. Becker, unpublished data). We note the Becker and Quang (2009) population estimate of 618.4 brown bears (26.3 brown bears/1,000 km^2^) is much lower than the MRDS_2PN model estimate of 746.1 (Table 2) due mainly to un‐modeled heterogeneity in the full independence model used by Becker and Quang (2009). Schmidt et al. (2017) are not justified in dropping the MR data since 2 of their reported MR probabilities were calculated using a full independence MRDS model (Becker & Quang, 2009) and, as a result, are biased high due to un‐modeled heterogeneity.

We applied the MRDS_2PN model to four brown bear datasets and obtained weighed mean mark–recapture probabilities that range from 0.828 to 0.952 (Table 1). The minimum of the individual mark–recapture estimates for these surveys range from 0.418 to 0.888. Black bear are generally more easily detected and have high MR probabilities (mean 0.926, minimum 0.644, Table 1, Becker & Christ, 2015). The Katmai brown bear dataset (2004, 2005) used by Quang (2005) to calculate an MRDS estimate under the full independence assumption (Becker & Quang, 2009) was re‐analyzed with the MRDS_2PN model, and the estimated apex MR estimate was 0.928 (*SE* = 0.020) with a range of 0.504 to 0.969 for individual estimates. This is the same survey (Katmai 2004, 2005) that Schmidt et al. (2017) claim has mark–recapture estimates so close to 1 to justify not using the mark–recapture data. These estimates indicate the perfect detection assumption is violated and MR data and modeling is needed. One might argue the uncertainty about the mean black bear MR estimate in Becker and Christ (2015) could justify assuming it is one, but a mean point estimate of 0.926 (*SE* = 0.038) is not close to 1 and the minimum individual estimate of 0.644 would indicate the survey methodology results in imperfect detection for some bears.

**Table 1:** Mark–recapture parameter estimates for five aerial bear surveys conducted in Alaska

Survey	Mean MR *p* (*SE*)	Range MR *p_i_*	Half‐normal *n* left‐truncations (%)
Southcentral Alaska, Black Bear (Becker & Christ, 2015)	0.926 (0.038)	0.644, 1.000	64 (27.2%)
Southcentral Alaska, Brown Bear (Becker & Quang, 2009)	0.828 (0.045)	0.800, 0.871	28 (18.3%)
Southern AK Peninsula Brown Bear (Becker unpublished data)	0.876 (0.022)	0.418, 0.955	37 (13.8%)
Unimak Island, Alaska (Becker unpublished data)	0.952 (0.041)	0.888, 0.983	13 (11.6%)
Katmai National Park (2004, 2005) (Quang, 2005)	0.928 (0.020)	0.504, 0.969	90 (19.8%)

The Alaska Department of Fish and Game has conducted seven MRDS surveys for bears; the survey with the most open habitat was a brown bear survey in the northern Brooks Range. That study area was so open, vegetative cover could not be considered as a covariate, due to very small sample sizes for percent cover values greater than 0. Surprisingly, the apex MR estimate was 0.794 (*SE* = 0.086) with a range of 0.253 to 0.936 for individual estimates, and is the lowest we have seen (E. Becker, unpublished data). This mark–recapture estimate indicates open terrain does not guarantee the perfect detection assumption is met for bear surveys using aircraft. Based on the results of MRDS modeling using the MRDS_2PN model, none of these seven surveys would meet the perfect detection assumption; further, that the results indicate MR data and modeling is needed to obtain population estimates. These surveys include three brown bear surveys conducted on the Alaska Peninsula, the geographic focus of Schmidt et al., 2017 (Table 1).

## 3 POOLING ROBUSTNESS

The models proposed by Schmidt et al. (2017) ignored covariates and thus avoided MCDS models by taking advantage of a property of CDS models called “pooling robustness” (Buckland et al., 2015). Under pooling robustness, CDS models are not prone to bias due to un‐modeled heterogeneity. However, there are three cases in which the property of pooling robustness fails: (a) if detection, for all bears, at the apex of detection, is not true (Buckland et al., 2015); (b) if the study design incorporates stratified sampling with unequal sample intensities (Buckland et al., 2015); and (c) if there are extreme levels of heterogeneity in levels of a covariate (Marques, Thomas, Fancy, & Buckland, 2007). The CDS_HBayes model violates case (b) because the pooled study areas are, in effect, strata for which separate estimates are desired and each has its own sample intensity. As previously documented, aerial surveys of brown and black bears in Alaska do not meet the perfect detection assumption [case (a)] which causes the failure of pooling robustness.

## 4 ESTIMATED RELATIVE BIAS

Using the apex of detection of the best fitting MRDS_2PN models for five bear surveys in Alaska would result in a left truncation of 11.6% to 27.2% of the data (Table 1) if a half‐normal distribution was used instead. Comparing CDS models using a half‐normal distribution with those of the best MRDS_2PN models to estimate relative bias would subject the results to the confounding effect of reduced sample size. In order to calculate only the relative bias from ignoring mark–recapture data and covariates, we compared the population estimate of a CDS model based on a 2‐piece normal distribution to the best MRDS_2PN model for five aerial bear surveys. We found that ignoring mark–recapture data and covariates resulted in a −17.4% to −21.4% bias compared to the MRDS_2PN models (Table 2). These results indicate the need for models, such as the MRDS_2PN model, that utilize covariates and mark–recapture data to obtain population estimates.

**Table 2:** Relative bias of conventional distance sampling (CDS) models as measured against MRDS_2PN models for five aerial bear surveys conducted in Alaska

Bear Survey	Change in AIC MRDS_2PN vs. CDS_2PN	CDS_2PN Population estimate	MRDS_2PN Population estimate	Relative bias of the CDS estimate	Apex distance MRDS_2PN model (m)
Southcentral Alaska, Black Bear (Becker & Christ, 2015)	−50.15	1,901.1	2,377.0	−20.02%	102.6
Southcentral Alaska, Brown Bear (Becker & Quang, 2009)	−8.72	586.6	746.1	−21.38%	112.3
Southern AK Peninsula Brown Bear (Becker unpublished data)	−24.82	1,431.5	1,764.7	−18.88%	110.6
Unimak Island, Alaska (Becker unpublished data)	−18.31	248.5	302.9	−17.96%	111.8
Katmai National Park (2004, 2005) (Quang, 2005)	−95.10	1,486.4	1,798.9	−17.37%	123.8

Notes

CDS_2PN Population Estimate—based on a CDS model using a two‐part normal detection function (Becker & Christ, 2015). MRDS_2PN Population Estimate—estimate obtained using the best MRDS_2PN model with covariates, where AIC is used to select the best model.

Relative Bias of the CDS Estimate—the bias is calculated relative to the MRDS_2PN model as follows: [(CDS Estimate − MRDS_2PN Estimate)/MRDS_2PN Estimate]%.

## 5 AVAILABILITY

Schmidt et al. (2017) raise concerns about all bears being available to be sampled and proposed an availability model to determine the extent of the problem. If a randomly placed transect can result in a bear being detected, then it is available to be detected by the sampling process. The only bears unavailable to be sampled are bears in dens; study areas with heavy canopy cover that preclude the observer seeing the ground are inappropriate for these surveys. These surveys are conducted just prior to leaf‐out to minimize or eliminate the problem of bears remaining in their dens during the survey. The basic premise of distance sampling is that the detection shape can be correctly estimated by the detection model and scaled correctly using either an assumption of perfect detection, or MR data to obtain the correct MR probabilities for estimating population size (Laake et al., 2008).

CDS and CDS_Hn models assume perfect detection at the apex. If this assumption is true and the detection function can be adequately modeled, then the availability of non‐den bears is not an issue, the population is estimated correctly (Buckland, 2001), and the complex CDS_OpenHnBayes model is not needed. Laake et al. (2008) state “as long as *p*(0) = 1, it does not matter why groups for *x* > 0 were missed.” Here, *p*(0) refers to the detection probability at the apex distance and x denotes distance. For MRDs models using point independence, Laake et al. (2008) point out that animals at the apex distance that are undetectable due to vegetation would be a source of bias. This is not availability bias if a randomly placed transect can detect the animal, but rather a bias in the MR model.

Laake et al. (2008) stated that in aerial surveys, animals become undetectable from the transect, due to cover and terrain not allowing the animal to be sighted at farther distances. Like Laake et al. (2008), we find this condition generally occurs at the farther distances in aerial distance sampling surveys. In aerial bear surveys, the detection apex distance usually occurs in the 100–125 m range (Table 2), so topography and vegetation at this distance would have to totally obscure the bear from every sighting angle for the bear to be a source of bias to the MR model. The 100–125 m distance range has good sighting angles, a sufficient height advantage, and sufficient time (as the plane flies by) to detect bears. For these reasons, the percentage of bears that are undetectable from the transect would be lowest at this distance.

We know of two instances of bears being missed in the approximate range of the apex distance and the circumstances were documented to determine if the bears were detectable. In the first instance, five bear groups with a total of 14 bears were observed from a transect that included a whale carcass on a beach. In circling, these bear groups to obtain GPS locations and enumerate group size, an additional bear group of 1 was observed that was not detected from the transect at an approximate distance of 175 m. This bear was visible from this transect but missed by both pilot and observer; it would be an undetected bear that was available to be detected. The second instance involves a transect along a straw‐colored, long‐grass mountainside with many dark trees lying on the ground from a past windstorm. This transect had gone up a valley and, after the transect was flown, the plane exited the valley by re‐flying the general route. At this time, a large, blond‐colored brown bear, with a distinctive dark stripe on its back, was observed lying down along the previously searched mountainside. The bear was easily observed, so the observer decided to line up the flight path on the computer with the GPS location of the plane and re‐fly the transect section to determine why the bear was missed. At sighting angles outside the 70‐ to 110 degree (approximately) angle, the blond bear was readily detected due to its bear like outline. Once the steeper angle threshold (70–110) was encountered, the bear outline vanished and it instantaneously “became a downed log” (due to the dark streak on its back) in a straw‐colored, long‐grass field. It was estimated that the distance to the bear was in the 100–150 m range. This detectable bear was probably missed by both pilot and observer searching the bear's location at search angles within the 70–110 degree range. This would be an undetected bear that was available to be detected. In the thousands of times, we have flown off transect to mark GPS locations of sighted bears or determine if a sighting was a bear, we have yet to encounter a situation where a bear was detected off transect at a distance associated with detection apexes and considered to be undetectable from the transect. Based on our experience, including those detailed above, we feel the number of undetectable bears at apex distances is 0 or very close to 0. Bears that are undetected at the apex distance are estimated using the MR model.

## 6 AVAILABILITY MODELING

Schmidt et al. (2017) propose the CDS_OpenHnBayes model to estimate p_a_ (the probability a bear is available to be detected given it is in the study area) and claim using this estimate on a CDS half‐normal model will correct for missed bears. The CDS_OpenHnBayes model is an open distance model with temporary immigration (Kery & Royle, 2016) which is based on an N‐mixture model that uses replicated count data (Royle, 2004), in this case survey days with different transects. Schmidt et al. (2017) list two assumptions; (a) population closure, and (b) “that the daily survey data adequately represented the study population available to be sampled each day.” They claim “variation in detection among survey dates would then reflect variation in *p*_a._” Additionally, they claim “under this formulation, incomplete detection of available bears, at the apex of detection would be incorporated into the availability parameter.” They also point out that this model obtains an estimate of the super population versus a population snap‐shot if the closure assumption is violated.

We disagree that these populations are closed; brown bears have large home ranges and there is no geographic barrier to their movements into or out of the Katmai study area. We also disagree that these survey days will be representative. Bear density within a study area is not uniform, some small locations or transects exhibit a super abundance of bears (“nugget effect”). The Unimak Island brown bear survey had one transect on the coast that had five bear groups detected on it, for a total of 14 bears; the reason was this transect searched an area that included a recently washed up whale carcass which was being scavenged by many bears. The Katmai study area has thin strips of coastal sedge meadows containing an important brown bear food source during a time when food is generally scarce. These locations are so coveted by brown bears that they look more like a cattle ranch versus general bear habitat. The Katmai dataset had one transect in this coastal sedge meadow habitat from which 13 bear groups were detected containing a total of 26 bears, the next highest transect had eight groups containing a total of 12 bears. These “nugget effects” can occur by random chance or for biological reasons like those listed above, and are too rare to occur daily. The net effect is to cast serious doubt on the assumption that survey days are representative for these surveys.

Additional assumption problems include the fact that not all assumptions are listed. One additional assumption is that availability of individuals is independent over time. Barker, Schofield, Link, and Sauer (2018) point out another assumption which they refer to as the “constant p assumption.” N‐mixture model (such as the CDS_OpenHnBayes model) has the assumption that individual detection probabilities are constant with respect to un‐modeled covariates (“constant p assumption”) (Barker et al., 2018).

Observers are often changed during the course of the survey; this will affect detection since the ability of observers is highly variable and, thus, availability will be confounded with observers and violate the assumption of the availability of individual bears is independent of time. Many observers and some pilots often gain a clearer bear search image after the first day or two of the survey, which would again confound bear availability with observer efficiency and thus violate the assumption of the availability of individual bears being independent of time. Differences in lighting can have a drastic impact on bear detection. On a day with good lighting, a bear in the shadows of a shrub patch of alders at 400 m can be easily detected. On a day with poorer light conditions, it is doubtful this bear can be detected at 400 m, in which case it would be classified as “temporarily unavailable” in the context of an availability model. If bears in dens exist during a survey, an availability model will not incorporate them into the population estimate. The availability of bears in dens is also not independent of time, because knowing a bear is in a den increases the likelihood it will remain in the den on the following day. As previously stated, these surveys are designed to minimize this unavailability bias of bears in dens. These issues would indicate that the availability of individual bears is not constant over time.

The use of covariates and mark–recapture data in our MRDS_2PN model indicates tremendous variability in bear detection probabilities with aerial distance sampling surveys. These detection probabilities vary due to covariates such as percent cover around the bear, search distance bin, bear activity, airplane speed, and others. Detection of bears will vary among individuals and also vary among multiple detections of the same bear on subsequent days. The CDS_OpenHnBayes model uses a constant detection probability, but aerial bear detections have been shown to differ due to many different covariates (Becker & Christ, 2015) which indicate these surveys cannot meet the “constant p assumption.” Additionally, there are covariates that affect detection but are very difficult to quantify and incorporate into a model. One such covariate would be lighting conditions which can greatly increase or decrease bear detection. The fact that not all covariates can be quantified makes it extremely doubtful that all heterogeneity can be entirely eliminated by modeling. Barker et al. (2018) conclude “fundamentally count data under imperfect detection can only reliably be used as indices.” In addition, they state that from “a mark‐recapture perspective, we show the loss of information that results from not marking animals is critical, making reliable statistical modeling of N and p problematic using just count data.” The use of covariates to model detection and MR probabilities and the presence of hard to quantify covariates indicate the sampling process contains heterogeneity in the detection probabilities. This heterogeneity results in the CDS_OpenHnBayes model being inappropriate for these surveys.

From figure 3b in Schmidt et al. (2017), the super‐population estimate is approximately 3,000 bears for the 2004–2005 Katmai bear survey. For that same survey, we report a population estimate of 1,798.9 bears (Table 2). The difference between population and super population may account for some of this difference. The most parsimonious explanation would be the assumptions of the CDS_OpenHnBayes model cannot be met by the aerial bear survey process and thus this model is inappropriate.

## 7 ADDITIONAL MRDS_2PN CONCERNS

Schmidt et al. (2017) stated that because of high correlation between effective search distance (ESD) (either binned or non‐binned) and distance, they did not include ESD in their analysis. The issue is not correlation but rather that MCDS models assume independence between the covariates and distance. Becker and Christ (2015) used 9 bins for the ESD covariate to try and minimize this problem. Lately, we have used three bins {[0–0.6*w], (0.6*w‐*w*], (>*w*)}, where *w* denotes the truncation distance; however, knowing ESD is in Bin 1 gives information on possible values for distance (*y*) since *y* ≤ ESD < 0.6*w* for this bin. For this reason, we now use two bins {[0‐*w*], (>*w*)} and by doing so, create an ESD covariate that is independent of distance. For the brown bear survey results given in Table 2, binned ESD covariate was created using two bins as outlined above. The survey results of the black bear survey are from Becker and Christ (2015) which we chose not to reanalyze in order to avoid confusion. Population estimates listed in Table 2 for the MRDS_2PN model are from the best covariate model, based on AIC criteria (Buckland et al., 2015).

Schmidt et al. (2017) state the MRDS_2PN model requires “much larger sample sizes (i.e., ≥150 detections)” than the 60–80 detections needed to fit a CDS model (Buckland et al., 2001). In fact, no minimum sample size requirements have ever been published for the MRDS_2PN model. For the Becker and Quang (2009) Gamma‐based MRDS model, 150 observations were recommended to obtain specified precision goals (Thompson, Peirce, & Mangipane, 2010). In terms of fitting a model, 70–100 detections should be sufficient for either method. MR models require less data than that for fitting a detection function; since the models are fit separately, sample size requirements would be based on the needs of fitting the detection function.

Encounter rate is an important component of the variance of distance sampling models (Fewster et al., 2009). Unfortunately, we misinterpreted the Borchers et al. (2006) paper and omitted this component from our r‐code (Becker & Christ, 2015). All models, including Bayesian models, should incorporate the encounter rate into the variance estimate.

## 8 DISCUSSION

Schmidt et al. (2017) took a total error approach (Reynolds, 2012) that allows for biased estimates to be considered. They reported very small bias (−9% to +2%) of the CDS_Hn population estimates to those from the Schmidt et al. (2017) created “Becker‐Christ approach.” The lack of covariates in the “Becker‐Christ approach” causes un‐modeled heterogeneity in MR probabilities resulting in positive bias in the probability of detection and negative bias in the population estimate, and thus a flawed MRDS model (Buckland et al., 2015). Buckland et al. (2015) do “not consider MRDS models with no covariates other than distance,” such as the “Becker‐Christ approach” due to the un‐modeled heterogeneity. A more appropriate model for this comparison would be the MRDS_2PN model (Becker & Christ, 2015) containing covariates which results in much larger bias (−17% to –21%, Table 2). For the KATM survey, they reported a bias of +2% and we report a bias of −17% (Table 2). Buckland et al. (2001) warn against selecting for models on the basis of precision at the expense of larger bias. They recommend that “the estimator be roughly unbiased, or at least that there is no reason to suppose it might be more biased than other robust estimators, before selecting on the basis of efficiency,” where efficiency denotes low variance. These recommendations would advocate for the selection of the MRDS_2PN model over the CDS‐Hn or CDS_HnBayes model.

Buckland et al. (2001) noted that “generally, as the number of parameters in a model increases the bias decreases but the sampling variance increases. A proper model should be supported by the particular dataset and thus have enough parameters to avoid large bias but not so many that precision is lost (the Principle of Parsimony). The relative fit of alternative models may be evaluated using Akaike's Information Criteria” (AIC). The “precision gains” of the CDS_Hn and CDS_HnBayes models over the “Becker‐Christ approach” is to be expected for three reasons. First, precision can be gained at the expense of bias (Buckland et al., 2001). Second, a flawed model, the “Becker‐Christ approach” model, was used in the comparison. Third, the “Becker‐Christ approach” model had three additional parameters (one for the two‐piece normal and two for the MR model). If AIC was used to evaluate CDS versus MCDS models, MCDS models would have been selected (Table 2). In addition to ignoring model selection, Schmidt et al. (2017) did not assess model fit, although evidence of model convergence was provided. Buckland et al. (2001, 2015) stress the importance of evaluating the model fit to the distance data. Burnham et al. (2004) advocate goodness of fit tests, *q*–*q* plots, the Kolmogorov–Smirnov test, and the Cramer–von Mises test as ways to evaluate the fit of the detection model.

The Becker and Christ (2015) MRDS model (MRDS_2PN) utilizes covariates while the “Becker‐Christ approach,” constructed by Schmidt et al. (2017) did not contain covariates. Since MRDS models rely on MR data and use an MR model they are not pooling robust, and as a result, it is very important to incorporate covariates into the model (Borchers et al., 2006; Buckland et al., 2015; Laake & Borchers, 2004). To date, aerial distance sampling of brown and black bears in Alaska has been unable to meet the assumption of perfect detection at the apex. We have demonstrated MR probabilities that are much smaller than 1 to backup this assertion. The perfect detection and pooling robustness assumptions of the CDS_HN and CDS_HnBayes models are violated by the current aerial bear survey methodology and these models are inappropriate for these surveys.

Distance sampling has been successfully used to estimate bear population size over large geographic areas (Becker & Christ, 2015). There are several ways to model this data. We view the availability modeling as having assumptions that are unlikely to be met by the current survey methodology. The assumptions associated with detection modeling using MRDS models seems more likely to be met by our current aerial surveys. Aerially collected distance sampling data have some unique properties that will drive the selection of the distance model. First, perfect detection cannot be assumed and an MRDS model will almost certainly be required. Second, the detection distance data are unimodal for these bear surveys, and either the gamma (Becker & Quang, 2009) or the two‐piece normal detection functions (Becker & Christ, 2015) are appropriate parametric models for this data. If one were willing to left truncate the data, a half‐normal detection function could be used. The use of a half‐normal detection model would result in less data (12%t to 27%, Table 1) for modeling but would result in a simpler detection model, one less parameter than the two‐piece normal detection function (Becker & Christ, 2015). A point independence MRDS model can be fit with the r‐package mrds (Laake, Borchers, Thomas, Miller, & Bishop, 2018) when a half‐normal detection function is specified. An MRDS model that uses the gamma detection function (Becker & Quang, 2009) cannot be used with the point independence assumption (Becker & Christ, 2015); for this reason we do not recommend this approach.

To model our aerial distance sampling data, we chose the MRDS_2PN model over an MRDS model that uses a half‐normal detection function for three reasons. First, we preferred the additional data (12% to 27%, Table 1) at the cost of only 1 additional parameter. Second, the use of density surface models (Hedley & Buckland, 2004; Miller, Burt, Rexstad, & Thomas, 2013), which model the results of the MRDS model to improve estimation precision, are complex models that benefit from using all available data. Third, the distance selected for left truncation is often subjective, but this location (the apex of detection) is the most important location for model fit (Buckland et al., 2001) since the population estimate is based on estimated animal density at this location (Buckland et al., 2001; Laake et al., 2008); whereas in the MRDS_2PN model, this location is estimated as a parameter. One could take the approach of Schmidt et al. (2017) and use a two‐piece normal model to estimate the apex location; unlike their approach which did not use covariates, a full model selection process among the various covariates would need to occur since the apex distance parameter changes with model selection. Our MRDS_2PN model minimizes left truncation data loss and the apex location is an estimated parameter while an MRDS model that uses a half‐normal detection function does not minimize data loss and often the left truncation distance is subjective.

We agree that the MRDS_2PN model is complicated but assert the additional components beyond those of a CDS model were needed to build models whose assumptions could be met by our current sampling scheme. We use the two‐piece normal detection function to avoid left truncation. Our analysis has documented the need for MR data and modeling. This in turn causes covariate modeling since pooling robustness does not hold in the absence of perfect detection. By applying the MR model only to the apex of detection (point independence), our models minimize the bias of impossible to detect bears at this distance because this is the best distance to detect bears. In contrast, a non‐point independence approach, like a full independence MRDS model would use all the MR data to scale the detection function resulting in a higher percentage of impossible to see bears causing bias in the estimate. Laake et al. (2008) make a strong argument that animals are hidden in aerial surveys due to vegetation and topography, and that this is more likely to occur at larger distances.

For management agencies, like the Alaska Department of Fish and Game, unbiased estimates of population size are very important because they allow hunter harvest to be directly compared to the population estimate to obtain estimates of hunter harvest rates. Estimates of hunter harvest rates are rarely available. When we can obtain them, they allow for a direct assessment of our harvest goals or desired population levels and help direct management changes if they are needed. The use of hunter harvest statistics with super‐population estimates would be very difficult to interpret.

## CONFLICT OF INTEREST

None declared.
